# Margin Integrity of Bulk-Fill Composite Restorations in Primary Teeth

**DOI:** 10.3390/ma13173802

**Published:** 2020-08-28

**Authors:** Alina Paganini, Thomas Attin, Tobias T. Tauböck

**Affiliations:** 1 Department of Orthodontics and Pediatric Dentistry, Center for Dental Medicine, University of Zurich, 8032 Zurich, Switzerland; alina.paganini@zzm.uzh.ch; 2 Department of Conservative and Preventive Dentistry, Center for Dental Medicine, University of Zurich, 8032 Zurich, Switzerland; thomas.attin@zzm.uzh.ch

**Keywords:** bulk-fill resin composite, biomaterials, deciduous dentition, thermo-mechanical loading, marginal adaptation

## Abstract

This in vitro study examined the margin integrity of sculptable and flowable bulk-fill resin composites in Class II cavities of primary molars. Standardized Class II cavities were prepared in human primary molars and restored with the following resin composite materials after application of a universal adhesive: a sculptable bulk-fill composite (Tetric EvoCeram Bulk Fill (TEC) or Admira Fusion x-tra (AFX)), a flowable bulk-fill composite (Venus Bulk Fill (VBF) or SDR), or a conventional composite (Filtek Supreme XTE (FS)). The bulk-fill materials were applied in 4 mm layers, while the conventional composite was applied in either 2 mm (FS2, positive control) or 4 mm layers (FS4, negative control). The specimens were exposed to thermo-mechanical loading (TML) in a computer-controlled masticator. A quantitative margin analysis was performed both before and after TML using scanning electron microscopy, and the percentage of continuous margins (margin integrity) was statistically analyzed (α = 0.05). All composites showed a significant decline in margin integrity after TML. AFX exhibited the significantly highest margin integrity of all materials after TML (97.5 ± 2.3%), followed by FS2 (79.2 ± 10.8%), TEC (73.0 ± 9.1%), and FS4 (71.3 ± 14.6%). SDR (43.6 ± 22.3%) and VBF (25.0 ± 8.5%) revealed the lowest margin integrity. In conclusion, the tested sculptable bulk-fill materials show similar or better margin integrity in primary molars than the conventional resin composite placed in 2 mm increments.

## 1. Introduction

Today, carious primary teeth are mostly restored by using tooth-colored filling materials such as glass ionomer cements, compomers, and resin-based composites [1]. Among these materials, the lowest failure rates have been reported for resin composites [2]. However, an inherent problem of resin composites is the fact that they shrink during the polymerization process, which leads to contraction stresses within the material and at the tooth–restoration interface [3]. As a result, margin integrity of composite restorations can be compromised, and microleakage, postoperative sensitivity, recurrent caries, and retention loss may occur [4,5].

Various incremental layering techniques and modified light curing approaches have been established to counteract the consequences of contraction stress [6,7]. However, these approaches are technique-sensitive which, in particular, is a problem when treating children. Furthermore, light curing several composite layers consecutively in deep cavities is time consuming and may lead to the incorporation of air bubbles or contaminants, especially when the compliance of a young patient is lacking [8].

To simplify and expedite restorative interventions, bulk-fill resin composites have been developed. These materials, which can be classified as low-viscosity (flowable) and high-viscosity (sculptable) bulk-fill composites, possess increased depths of cure compared with conventional resin composite materials, allowing placement and photopolymerization of thick composite layers up to 4–5 mm [9,10,11]. Both flowable and sculptable bulk-fill composites have been found to generate lower shrinkage forces [12,13,14] and less cuspal flexure [15,16] than conventional resin composites. Furthermore, similar marginal adaptation [17,18,19] and similar clinical success rates [20,21,22] of bulk-fill and conventional composites have been reported in permanent teeth. A recent study showed significantly lower microleakage of bulk-filled proximal cavities of permanent teeth with gingival margins located in the enamel than in dentin [23]. In addition, the marginal sealing ability of bulk-fill composites has been revealed to depend on the specific material used [24]. While marginal sealing properties of bulk-fill composites have been extensively studied in permanent teeth [17,18,19,23,24], only scarce information is available for primary teeth [25] and, to date, the feasibility of the application of flowable and sculptable bulk-fill composites in primary teeth has not been comprehensively investigated. Thus, research on the margin quality of these materials in primary teeth is highly required.

Based on these considerations, the aim of this in vitro study was to evaluate in primary molars the margin integrity of resin composite restorations made of bulk-fill composite materials applied in 4 mm layers, or of conventional composite material, both before and after thermo-mechanical loading, in an artificial mouth environment. The null hypothesis to be tested was that there would be no significant differences in margin integrity between cavities restored with conventional or bulk-fill composite materials.

## 2. Materials and Methods

### 2.1. Specimen Preparation

Thirty extracted, non-carious human primary molars stored in 0.1% thymol solution were chosen for this in vitro study. There was always a therapeutic indication for tooth extraction, e.g., an ankylosis of the root or orthodontic reasons. Only extracted teeth from patients that gave written, informed consent prior to the further use of their extracted teeth for research purposes were included, and the teeth were irreversibly anonymized immediately after extraction. Under these terms, the research complied with the use of anonymized biological material and, therefore, authorization from the ethics committee was not required (BASEC request-no. 2018-00317).

**Figure 1:**
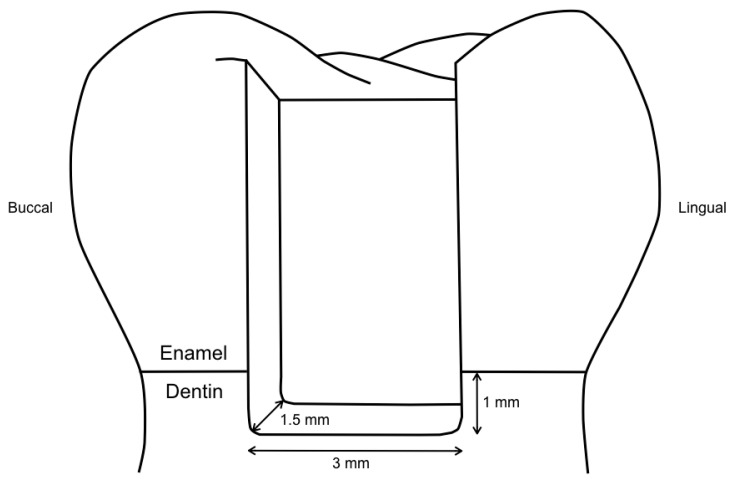
Illustration of the cavity design, with cervical margins 1 mm below the cementoenamel junction in dentin.

After cleaning the teeth of plaque, gingival tissue, and calculus, standardized Class II cavities were prepared mesially and distally in each tooth, using a high-speed contra-angle handpiece (Sirius, Micro-Mega, Besançon Cedex, France) rotating at 180,000 rpm. The dimensions of the cavities were 3 mm in width and 1.5 mm in depth, with cervical margins 1 mm below the cementoenamel junction in dentin (Figure 1), as checked with a periodontal probe. Preparation was performed using 80 μm cylindrical diamond burs (Universal Prep Set, Intensiv, Grancia, Switzerland). Enamel margins were beveled with 40 μm flame-shaped diamond burs (Intensiv). All burs were exchanged after they had been used for the preparation of four cavities.

**Figure 2:**
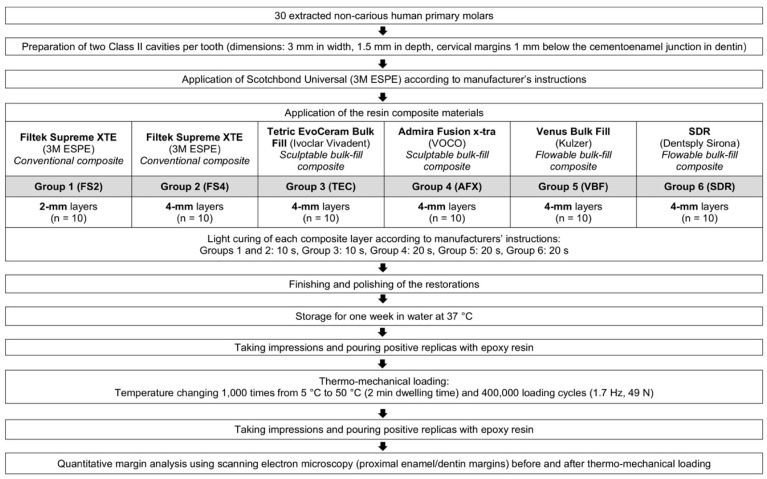
Experimental design.

Transparent matrices (Lucifix Matrix System, Kerr, Orange, CA, USA) were adapted around each tooth. Thereafter, all specimens were treated with a universal adhesive (Scotchbond Universal, 3M ESPE, St. Paul, MN, USA, LOT: 80426B) applied in self-etch mode according to the manufacturer’s instructions, by rubbing in for 20 s, gently air drying for 5 s, and light curing for 10 s at 1200 mW/cm^2^ with an LED curing unit (Bluephase G2, Ivoclar Vivadent, Schaan, Liechtenstein). The cavities (n = 10 per group) were then restored according to their experimental group, as detailed in Figure 2.

Group FS2 was incrementally filled with the conventional composite Filtek Supreme XTE (3M ESPE, St. Paul, MN, USA), with 2 mm thick horizontal layers (last increment for the residual height of the cavity ≤ 2 mm). All other groups received a first increment of 4 mm thickness and a second increment of ≤ 4 mm thickness for the residual height of the cavity, either with the conventional composite Filtek Supreme XTE (3M ESPE) (group FS4, negative control), or with the bulk-fill composites Tetric EvoCeram Bulk Fill (Ivoclar Vivadent, Schaan, Liechtenstein) (group TEC), Admira Fusion x-tra (VOCO, Cuxhaven, Germany) (group AFX), Venus Bulk Fill (Kulzer, Hanau, Germany) (group VBF), or SDR (Dentsply Sirona, Konstanz, Germany) (group SDR). The composition and manufacturers’ details of the composite materials are given in Table 1. Each increment was photoactivated with the Bluephase G2 light curing unit (Ivoclar Vivadent), following the manufacturers’ instructions (Figure 2). All restorations were finished and polished using Sof-Lex disks with decreasing grit sizes (Sof-Lex Pop-on, 3M ESPE, St. Paul, MN, USA) under continuous water cooling.

### 2.2. Thermo-Mechanical Loading

**Figure 3:**
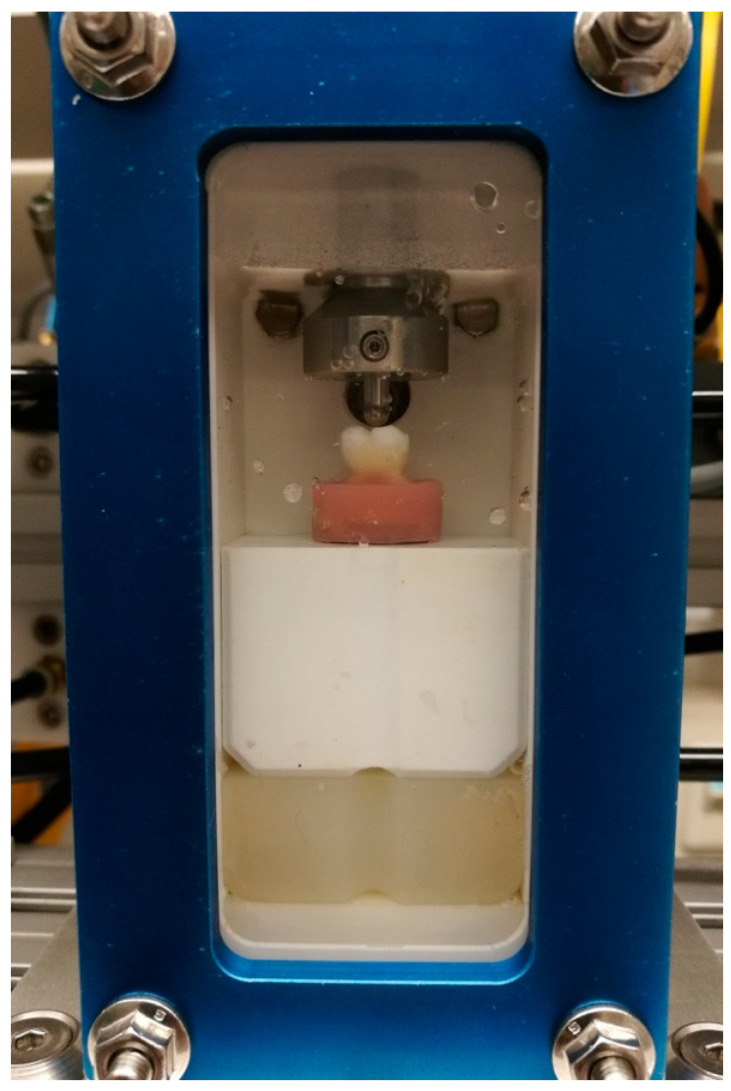
Test chamber of the computer-controlled masticator used for thermo-mechanical loading of the specimens.

After a one-week storage in water at 37 °C in the dark, the restored primary teeth were subjected to thermo-mechanical loading in a computer-controlled masticator (CoCoM 2, PPK, Zurich, Switzerland) (Figure 3) [26,27,28]. Thermocycling was carried out in flushing water, with 1000 temperature changes from 5 °C to 50 °C (2 min dwelling time). Mechanical stress was applied simultaneously, with a total of 400,000 loading cycles at 1.7 Hz and a load force of 49 N, using standardized metal balls with a diameter of 1.4 mm as antagonists.

### 2.3. Assessment of Margin Integrity

**Figure 4:**
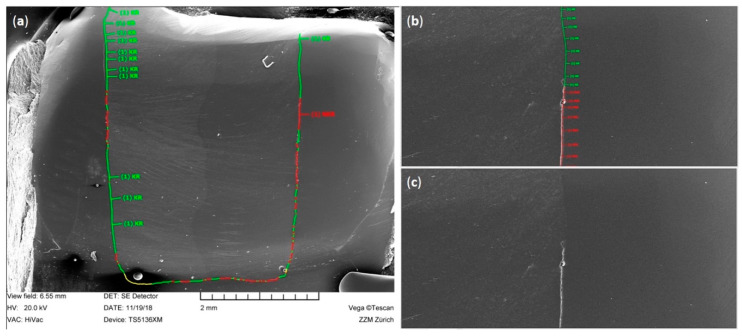
Illustration of the quantitative SEM margin analysis performed in this study. (**a**) SEM image of the entire proximal margin with classified continuous (green), non-continuous (red), and not judgeable (yellow) margin segments; (**b**) detail of image (**a**) with classified margin segments at 200 × magnification; (**c**) same image as (**b**) without labeled margin segments.

Before and after thermo-mechanical loading, impressions of the restorations were obtained using A-silicone (President Light Body, Coltène Whaledent, Altstätten, Switzerland). Thereafter, the impressions were poured out with epoxy resin (Epoxyharz L, R&G Faserverbundwerkstoffe, Waldenbuch, Germany) and glued on aluminum carriers (Cementit universal, Merz&Benteli, Niederwangen, Switzerland). After sputter coating the positive replicas with gold (Sputter SCD 030, Balzers Union, Balzers, Liechtenstein), they were subjected to a quantitative margin analysis using a scanning electron microscope (SEM) (Amray 1810/T, Amray, Bedford, MA, USA). The tooth–composite interface was classified as continuous, non-continuous, or not judgeable at 20 kV and 200x magnification, both before and after thermo-mechanical loading. The margin integrity was expressed as a percentage of the continuous margins for the total judgeable margin length, and also separately for enamel and dentin margins [28,29,30]. Figure 4 illustrates the method of the performed quantitative margin analysis.

### 2.4. Statistical Analysis

Non-parametric Kruskal–Wallis tests and Conover post-hoc tests, with p-values adjusted for multiple testing according to Holm, were used to analyze differences in margin integrity between experimental groups. Wilcoxon signed-rank tests were computed to detect differences in margin integrity before and after thermo-mechanical loading. Statistical analysis was carried out with the statistical software R [31], including the package ggplot2 [32], at a pre-set level of significance of α = 0.05.

## 3. Results

**Figure 5:**
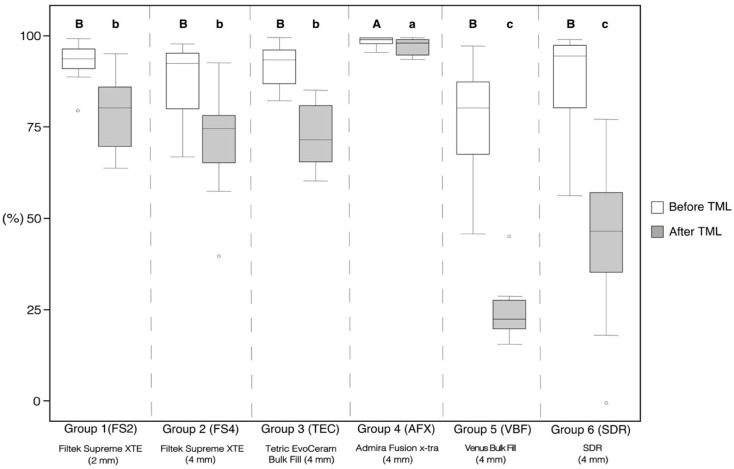
Margin integrity for the total margin length, given as percentages of continuous margins for all experimental groups before and after thermo-mechanical loading (TML). The boxplots represent the medians (black lines) with 25% and 75% quartiles (boxes). The whiskers represent 1.5*IQR (interquartile range), or minima and maxima of the distribution if below 1.5*IQR. Outliers are shown as circles. Statistically significant differences (*p* < 0.05) in the percentage of continuous margins between different groups are marked with different letters (capital and lower-case letters refer to the situation before and after TML, respectively). All groups showed a significant decline in margin integrity after TML (*p* < 0.05, respectively).

The percentages of continuous margins (margin integrity) considering the total margin length of all tested groups before and after thermo-mechanical loading (TML) are presented in Figure 5. Both before and after TML, AFX showed the significantly highest margin integrity of all groups (*p* < 0.05, respectively). The groups FS2, FS4, TEC, VBF, and SDR showed similar margin integrities before TML (*p* > 0.05, respectively). TML caused a significant decrease in margin integrity within all experimental groups (*p* < 0.05, respectively). After TML, the flowable bulk-fill resin composites VBF and SDR showed significantly lower margin integrities compared with the other groups (*p* < 0.001, respectively).

The results of the regional enamel and dentin margin integrity are given in Table 2. In enamel, AFX showed significantly higher margin integrity compared with VBF before TML (*p* < 0.001). After TML, AFX presented the significantly highest margin integrity of all groups (*p* < 0.05, respectively). FS2, FS4, and TEC showed no significant differences in margin integrity in enamel between each other after TML, but significantly higher values compared with VBF and SDR (*p* < 0.05, respectively). 

**Figure 6:**
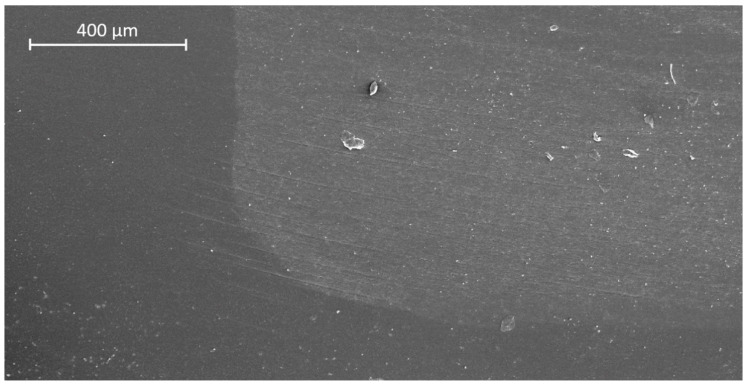
SEM image of continuous margin in dentin (group 4: Admira Fusion x-tra (AFX), after thermo-mechanical loading).

**Figure 7:**
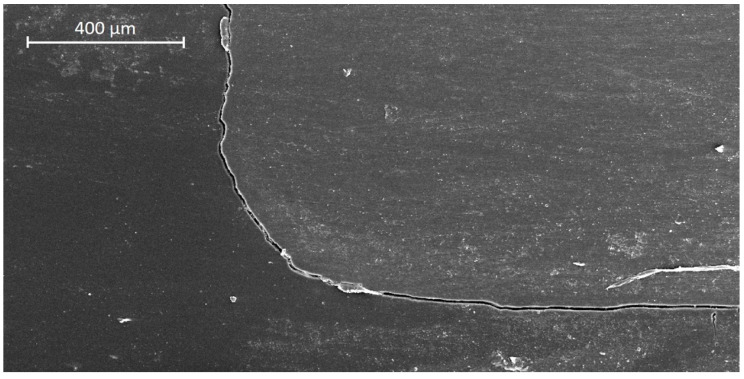
SEM image of non-continuous margin in dentin (group 5: Venus Bulk Fill (VBF), after thermo-mechanical loading).

In dentin, AFX showed significantly higher margin integrity before TML compared with FS4, TEC, VBF, and SDR (*p* < 0.05, respectively), but not compared with FS2 (Table 2). After TML, AFX obtained the significantly highest dentin margin integrity of all groups (*p* < 0.05, respectively), while VBF and SDR showed the lowest margin integrity. TML significantly deteriorated margin integrity within all experimental groups (*p* < 0.05, respectively), except for AFX in dentin. Representative SEM images of continuous and non-continuous margins are presented in Figure 6 and Figure 7, respectively.

## 4. Discussion

The results of this in vitro study show that the tested high-viscosity bulk-fill resin composites placed in 4 mm increments achieve similar or even higher margin integrities than a conventional composite placed in 2 mm increments, while the flowable bulk-fill composites under investigation revealed significantly lower margin integrities. Thus, the null hypothesis was rejected.

Bulk-fill composites are increasingly popular among dentists because they allow easier application techniques compared with conventional composites at similarly high clinical success rates [20,21]. Particularly in pediatric dentistry, the use of bulk-fill composites seems useful, since they facilitate a less technique-sensitive and less time-consuming filling therapy. In the deciduous dentition, other than in the permanent dentition, flowable bulk-fill composites do not need to be capped with a conventional hybrid composite. Thus, in the present study, no capping layer was placed on top of the flowable bulk-fill materials under investigation. The universal adhesive Scotchbond Universal was selected because it showed reliable results in both laboratory [33,34,35] and clinical studies [36,37]. To ensure time-saving application in the treatment of children, the universal adhesive was used in self-etch mode and thus was in line with previous studies [38,39]. A limitation of the present study is that only one adhesive was tested. The results can therefore not be generalized to other adhesives. Furthermore, in addition to margin integrity, other material parameters such as wear resistance, biocompatibility, and physico-mechanical properties also determine the clinical success of dental restorations [40,41,42,43,44], but were not evaluated in the present study.

As previously established, discontinuous filling margins may lead to secondary caries or retention loss [5,45]. Gap formation at tooth–restoration interfaces can be due to cyclic loading during chewing [46]. Furthermore, interfacial gaps may develop as a consequence of shrinkage stresses if the shrinkage forces of the resin composite exceed the tooth–composite bond strength [47]. Previous studies revealed lower shrinkage stress formation of flowable and sculptable bulk-fill composites compared with their conventional counterparts which, together with increased curing light transmittance, allows placement of bulk-fill composites in thicker increments [12,14,48]. In order to minimize shrinkage forces, manufacturers integrated in their bulk-fill composites proprietary base monomers with high molecular weights, partially functionalized fillers as shrinkage stress relievers, and unique stress-relaxant modulators [49]. In the present study, the high-viscosity bulk-fill composites under investigation showed higher margin integrities compared with the flowable ones. As a consequence of their higher filler content, high-viscosity bulk-fill composites have been shown to contract less during polymerization compared with flowable materials and to develop lower shrinkage stress at adhesive interfaces [14], which might have resulted in better marginal adaptation. Furthermore, the ormocer-based bulk-fill composite Admira Fusion x-tra revealed the highest percentage of continuous enamel and dentin margins, both before and after thermo-mechanical loading. A recent study examined the shrinkage force development of various bulk-fill and conventional composites and revealed that ormocer-based Admira Fusion x-tra induced the lowest stresses of all materials under investigation [14]. Its ormocer matrix differs chemically from conventional resin systems, since it contains inorganic–organic copolymers instead of classical monomers and a smaller organic resin amount compared with dimethacrylate-based composites, reducing volumetric contraction and shrinkage stress development [50,51,52,53]. In addition, the ormocer material has revealed favorable polymerization kinetics with a delayed onset of shrinkage stress generation, allowing the polymer network to partly relieve polymerization-induced shrinkage forces by viscous flow and molecular relaxation during the early stage of the polymerization reaction [14,54].

In the present study, the flowable bulk-fill composites SDR and Venus Bulk Fill demonstrated the lowest margin integrity of all materials after thermo-mechanical loading. As previously established, flowable bulk-fill materials possess inferior mechanical properties, such as lower surface hardness and lower modulus of elasticity compared with high-viscosity composites, which might compromise their long-term stability [41,55,56]. Furthermore, it has been shown that flowable bulk-fill composites, in particular SDR and Venus Bulk Fill, have a relatively low crosslinking density, resulting in a polymer network that can be more easily penetrated by solvents and allows for higher hygroscopic expansion [41,57,58]. Increased hydrolytic degradation of these materials over time may therefore have contributed to the observed inferior margin integrity [57,59].

The marginal adaptation of bulk-fill composites in permanent teeth has been extensively investigated. In accordance with our findings in primary teeth, previous studies in the permanent dentition demonstrated that the margin integrities of sculptable bulk-fill composites are comparable to conventional resin composites [19,30]. However, in contrast to the findings of the present research, flowable bulk-fill materials also attained similar margin integrities in permanent teeth as conventional, incrementally applied composites [17,18]. Furthermore, a recent systematic review and meta-analysis revealed no significant differences in enamel and dentin margin integrity between flowable and sculptable bulk-fill composites used for Class II restorations in the permanent dentition [60]. Other than in the aforementioned studies, in the present investigation, the flowable bulk-fill composites were not capped with a conventional hybrid resin composite in accordance with the manufacturers’ indications for the restoration of primary teeth. The omission of a capping made of a conventional composite might explain the observed inferior marginal integrity of the flowable materials, especially after thermo-mechanical loading. Future studies should therefore evaluate whether flowable bulk-fill composites would benefit from an applied capping layer when restoring primary teeth. This approach would, however, extend treatment time and render the filling procedure technically more demanding.

## 5. Conclusions

Within the limitations of the present in vitro study, the following conclusions can be drawn: (1) the tested sculptable bulk-fill materials show similar or better margin integrities in primary molars than a conventional, incrementally placed resin composite, (2) flowable bulk-fill composites applied in 4 mm thickness seem less suitable to restore primary teeth, due to their inferior sealing ability of enamel and dentin margins after thermo-mechanical loading, and (3) the ormocer-based bulk-fill composite provided the overall highest percentage of continuous enamel and dentin margins of all materials investigated.

## Figures and Tables

**Table 1:** Manufacturers’ information on the composite materials used in this study.

Composite	Manufacturer	LOT	Composition	Filler Content (wt%/vol%)
Filtek Supreme XTE	3M ESPE, St. Paul, MN, USA	N959768	Matrix: Bis-GMA, Bis-EMA, UDMA, TEGDMA, PEGDMA Filler: Silica/zirconia filler	78.5/63.3
Tetric EvoCeram Bulk Fill	Ivoclar Vivadent, Schaan, Liechtenstein	W07641	Matrix: Bis-GMA, Bis-EMA, UDMA Filler: Ba-Al-Si-glass, YbF_3_, spherical mixed oxide, PPF (monomer, glass filler and ytterbium fluoride)	81 (including 17% PPF)/61
Admira Fusion x-tra	VOCO, Cuxhaven, Germany	1824538	Matrix: Ormocer matrix Filler: SiO_2_, glass ceramics	84/69
Venus Bulk Fill	Heraeus Kulzer, Hanau, Germany	K010207	Matrix: UDMA, Bis-EMA Filler: Ba-Al-F-Si-glass, SiO_2_, YbF_3_	65/38
SDR	Dentsply Sirona, Konstanz, Germany	1806000680	Matrix: Modified UDMA, Bis-EMA, TEGDMA Filler: Ba-Al-F-B-Si-glass, Sr-Al-F-Si-glass	68/45

Bis-GMA: bisphenol-A-glycidyldimethacrylate; Bis-EMA: ethoxylated bisphenol-A-dimethacrylate; UDMA: urethane dimethacrylate; TEGDMA: triethylene glycol dimethacrylate; PEGDMA: polyethylene glycol dimethacrylate; PPF: prepolymer filler.

**Table 2:** Margin integrity, separately in enamel and dentin, given as percentages of continuous margins (mean ± standard deviation) for all experimental groups before and after thermo-mechanical loading (TML).

	Enamel		Dentin	
	Before TML	After TML	Before TML	After TML
Group 1: **FS2**	92.5 (5.9) ^AB^	85.3 (8.4) ^B^	94.9 (5.8) ^AB^	69.5 (21.1) ^B^
Group 2: **FS4**	89.5 (10.1) ^AB^	76.9 (13.5) ^B^	85.6 (20.0) ^B^	62.4 (27.6) ^BC^
Group 3: **TEC**	93.0 (6.5) ^AB^	82.6 (10.2) ^B^	91.0 (8.7) ^B^	59.0 (19.5) ^BC^
Group 4: **AFX**	98.1 (2.3) ^A^	97.3 (3.1) ^A^	100.0 (0.0) ^A^	97.7 (4.8) ^A^
Group 5: **VBF**	74.6 (21.9) ^B^	32.6 (16.1) ^C^	84.0 (12.0) ^B^	16.5 (8.4) ^D^
Group 6: **SDR**	90.7 (11.3) ^AB^	48.1 (29.6) ^C^	84.5 (18.8) ^B^	35.7 (29.4) ^CD^

Means followed by same superscript letters per column are not significantly different from each other at the 0.05 level. All groups, except Admira Fusion x-tra (AFX) in dentin, showed a significant decline in margin integrity after TML (*p* < 0.05, respectively).

## References

[1] Lazaridou D., Belli R., Krämer N., Petschelt A., Lohbauer U. (2015). Dental materials for primary dentition: Are they suitable for occlusal restorations? A two-body wear study. Eur. Arch. Paediatr. Dent..

[2] Chisini L.A., Collares K., Cademartori M.G., de Oliveira L.J.C., Conde M.C.M., Demarco F.F., Corrêa M.B. (2018). Restorations in primary teeth: A systematic review on survival and reasons for failures. Int. J. Paediatr. Dent..

[3] Davidson C.L., Feilzer A.J. (1997). Polymerization shrinkage and polymerization shrinkage stress in polymer-based restoratives. J. Dent..

[4] Carvalho R.M., Pereira J.C., Yoshiyama M., Pashley D.H. (1996). A review of polymerization contraction: The influence of stress development versus stress relief. Oper. Dent..

[5] Peutzfeldt A., Asmussen E. (2004). Determinants of in vitro gap formation of resin composites. J. Dent..

[6] Park J., Chang J., Ferracane J., Lee I.B. (2008). How should composite be layered to reduce shrinkage stress: Incremental or bulk filling?. Dent. Mater..

[7] Tauböck T.T., Feilzer A.J., Buchalla W., Kleverlaan C.J., Krejci I., Attin T. (2014). Effect of modulated photo-activation on polymerization shrinkage behavior of dental restorative resin composites. Eur. J. Oral Sci..

[8] Tarle Z., Attin T., Marovic D., Andermatt L., Ristic M., Tauböck T.T. (2015). Influence of irradiation time on subsurface degree of conversion and microhardness of high-viscosity bulk-fill resin composites. Clin. Oral Investig..

[9] Alrahlah A., Silikas N., Watts D.C. (2014). Post-cure depth of cure of bulk fill dental resin-composites. Dent. Mater..

[10] Tauböck T.T., Marovic D., Zeljezic D., Steingruber A.D., Attin T., Tarle Z. (2017). Genotoxic potential of dental bulk-fill resin composites. Dent. Mater..

[11] Dieckmann P., Mohn D., Zehnder M., Attin T., Tauböck T.T. (2019). Light transmittance and polymerization of bulk-fill composite materials doped with bioactive micro-fillers. Materials.

[12] Ilie N., Hickel R. (2011). Investigations on a methacrylate-based flowable composite based on the SDR technology. Dent. Mater..

[13] Marovic D., Tauböck T.T., Attin T., Panduric V., Tarle Z. (2015). Monomer conversion and shrinkage force kinetics of low-viscosity bulk-fill resin composites. Acta Odontol. Scand..

[14] Tauböck T.T., Jäger F., Attin T. (2019). Polymerization shrinkage and shrinkage force kinetics of high- and low-viscosity dimethacrylate- and ormocer-based bulk-fill resin composites. Odontology.

[15] Moorthy A., Hogg C.H., Dowling A.H., Grufferty B.F., Benetti A.R., Fleming G.J. (2012). Cuspal deflection and microleakage in premolar teeth restored with bulk-fill flowable resin-based composite base materials. J. Dent..

[16] Politi I., McHugh L.E.J., Al-Fodeh R.S., Fleming G.J.P. (2018). Modification of the restoration protocol for resin-based composite (RBC) restoratives (conventional and bulk fill) on cuspal movement and microleakage score in molar teeth. Dent. Mater..

[17] Roggendorf M.J., Kramer N., Appelt A., Naumann M., Frankenberger R. (2011). Marginal quality of flowable 4-mm base vs. conventionally layered resin composite. J. Dent..

[18] De Assis F.S., Lima S.N., Tonetto M.R., Bhandi S.H., Pinto S.C., Malaquias P., Loguercio A.D., Bandéca M.C. (2016). Evaluation of bond strength, marginal integrity, and fracture strength of bulk- vs. incrementally-filled restorations. J. Adhes. Dent..

[19] Heintze S.D., Monreal D., Peschke A. (2015). Marginal quality of Class II composite restorations placed in bulk compared to an incremental technique: Evaluation with SEM and stereomicroscope. J. Adhes. Dent..

[20] Van Dijken J.W.V., Pallesen U. (2017). Bulk-filled posterior resin restorations based on stress-decreasing resin technology: A randomized, controlled 6-year evaluation. Eur. J. Oral Sci..

[21] Heck K., Manhart J., Hickel R., Diegritz C. (2018). Clinical evaluation of the bulk fill composite QuiXfil in molar class I and II cavities: 10-year results of a RCT. Dent. Mater..

[22] Veloso S.R.M., Lemos C.A.A., de Moraes S.L.D., do Egito Vasconcelos B.C., Pellizzer E.P., de Melo Monteiro G.Q. (2019). Clinical performance of bulk-fill and conventional resin composite restorations in posterior teeth: A systematic review and meta-analysis. Clin. Oral Investig..

[23] García Marí L., Climent Gil A., LLena Puy C. (2019). In vitro evaluation of microleakage in Class II composite restorations: High-viscosity bulk-fill vs. conventional composites. Dent. Mater. J..

[24] Patel M.C., Bhatt R.K., Makwani D.A., Dave L.D., Raj V.S. (2018). Comparative evaluation of marginal seal integrity of three bulk-fill composite materials in Class II cavities: An in vitro study. Adv. Hum. Biol..

[25] Eltoum N.A., Bakry N.S., Talaat D.M., Elshabrawy S.M. (2019). Microleakage evaluation of bulk-fill composite in class II restorations of primary molars. Alex. Dent. J..

[26] Krejci I., Reich T., Lutz F., Albertoni M. (1990). An in vitro test procedure for evaluating dental restoration systems. 1. A computer-controlled mastication simulator. Schweiz. Monatsschr. Zahnmed..

[27] Krejci I., Planinic M., Stavridakis M., Bouillaguet S. (2005). Resin composite shrinkage and marginal adaptation with different pulse-delay light curing protocols. Eur. J. Oral Sci..

[28] Groddeck S., Attin T., Tauböck T.T. (2017). Effect of cavity contamination by blood and hemostatic agents on marginal adaptation of composite restorations. J. Adhes. Dent..

[29] Frankenberger R., Hehn J., Hajtó J., Krämer N., Naumann M., Koch A., Roggendorf M.J. (2013). Effect of proximal box elevation with resin composite on marginal quality of ceramic inlays in vitro. Clin. Oral Investig..

[30] Campos E.A., Ardu S., Lefever D., Jasse F.F., Bortolotto T., Krejci I. (2014). Marginal adaptation of class II cavities restored with bulk-fill composites. J. Dent..

[31] R Core Team (2015). R: A Language and Environment for Statistical Computing.

[32] Wickham H. (2016). ggplot2: Elegant Graphics for Data Analysis.

[33] Wagner A., Wendler M., Petschelt A., Belli R., Lohbauer U. (2014). Bonding performance of universal adhesives in different etching modes. J. Dent..

[34] Fu J., Saikaew P., Kawano S., Carvalho R.M., Hannig M., Sano H., Selimovic D. (2017). Effect of air-blowing duration on the bond strength of current one-step adhesives to dentin. Dent. Mater..

[35] Michelotti G., Niedzwiecki M., Bidjan D., Dieckmann P., Deari S., Attin T., Tauböck T.T. (2020). Silane effect of universal adhesive on the composite–composite repair bond strength after different surface pretreatments. Polymers.

[36] Lawson N.C., Robles A., Fu C.C., Lin C.P., Sawlani K., Burgess J.O. (2015). Two-year clinical trial of a universal adhesive in total-etch and self-etch mode in non-carious cervical lesions. J. Dent..

[37] Zanatta R.F., Silva T.M., Esper M., Bresciani E., Gonçalves S., Caneppele T. (2019). Bonding performance of simplified adhesive systems in noncarious cervical lesions at 2-year follow-up: A double-blind randomized clinical trial. Oper. Dent..

[38] Lenzi T.L., Pires C.W., Soares F.Z.M., Raggio D.P., Ardenghi T.M., de Oliveira Rocha R. (2017). Performance of universal adhesive in primary molars after selective removal of carious tissue: An 18-month randomized clinical trial. Pediatr. Dent..

[39] Papadogiannis D., Dimitriadi M., Zafiropoulou M., Gaintantzopoulou M.D., Eliades G. (2019). Universal adhesives: Setting characteristics and reactivity with dentin. Materials.

[40] Wegehaupt F.J., Tauböck T.T., Attin T. (2013). Durability of the anti-erosive effect of surfaces sealants under erosive abrasive conditions. Acta Odontol. Scand..

[41] Leprince J.G., Palin W.M., Vanacker J., Sabbagh J., Devaux J., Leloup G. (2014). Physico-mechanical characteristics of commercially available bulk-fill composites. J. Dent..

[42] Wegehaupt F.J., Tauböck T.T., Attin T., Belibasakis G.N. (2014). Influence of light-curing mode on the cytotoxicity of resin-based surface sealants. BMC Oral Health.

[43] Wiegand A., Credé A., Tschammler C., Attin T., Tauböck T.T. (2017). Enamel wear by antagonistic restorative materials under erosive conditions. Clin. Oral Investig..

[44] Par M., Spanovic N., Tauböck T.T., Attin T., Tarle Z. (2019). Degree of conversion of experimental resin composites containing bioactive glass 45S5: The effect of post-cure heating. Sci. Rep..

[45] Totiam P., Gonzalez-Cabezas C., Fontana M.R., Zero D.T. (2007). A new in vitro model to study the relationship of gap size and secondary caries. Caries Res..

[46] Aggarwal V., Logani A., Jain V., Shah N. (2008). Effect of cyclic loading on marginal adaptation and bond strength in direct vs. indirect class II MO composite restorations. Oper. Dent..

[47] Correia A., Andrade M.R., Tribst J., Borges A., Caneppele T. (2020). Influence of bulk-fill restoration on polymerization shrinkage stress and marginal gap formation in Class V restorations. Oper. Dent..

[48] Bucuta S., Ilie N. (2014). Light transmittance and micro-mechanical properties of bulk fill vs. conventional resin based composites. Clin. Oral Investig..

[49] Manhart J., Hickel R. (2014). Bulk-fill-composites. Modern application technique of direct composites for posterior teeth. Swiss Dent. J..

[50] Wolter H., Storch W., Ott H. (1994). New inorganic/organic copolymers (ORMOCERs) for dental applications. Mater. Res. Soc. Symp. Proc..

[51] Pick B., Pelka M., Belli R., Braga R.R., Lohbauer U. (2011). Tailoring of physical properties in highly filled experimental nanohybrid resin composites. Dent. Mater..

[52] Sharma S., Padda B.K., Choudhary V. (2012). Comparative evaluation of residual monomer content and polymerization shrinkage of a packable composite and an ormocer. J. Conserv. Dent..

[53] Rullman I., Patyna M., Janssen B., Willershausen B. (2017). Determination of polymerization shrinkage of different composites using a photoelastic method. Am. J. Dent..

[54] Lu H., Stansbury J.W., Bowman C.N. (2004). Towards the elucidation of shrinkage stress development and relaxation in dental composites. Dent. Mater..

[55] Ilie N., Bucuta S., Draenert M. (2013). Bulk-fill resin-based composites: An in vitro assessment of their mechanical performance. Oper. Dent..

[56] Par M., Marovic D., Attin T., Tarle Z., Tauböck T.T. (2020). The effect of rapid high-intensity light-curing on micromechanical properties of bulk-fill and conventional resin composites. Sci. Rep..

[57] Ferracane J.L. (2006). Hygroscopic and hydrolytic effects in dental polymer networks. Dent. Mater..

[58] Alshali R.Z., Salim N.A., Satterthwaite J.D., Silikas N. (2015). Post-irradiation hardness development, chemical softening, and thermal stability of bulk-fill and conventional resin-composites. J. Dent..

[59] Lima A.F., Soares G.P., Vasconcellos P.H., Ambrosano G.M., Marchi G.M., Lovadino J.R., Aguiar F.H. (2011). Effect of surface sealants on microleakage of Class II restorations after thermocycling and long-term water storage. J. Adhes. Dent..

[60] Gerula-Szymańska A., Kaczor K., Lewusz-Butkiewicz K., Nowicka A. (2020). Marginal integrity of flowable and packable bulk fill materials used for class II restorations–A systematic review and meta-analysis of in vitro studies. Dent. Mater. J..

